# Development of a Sodium Alginate-Based Active Package with Controlled Release of Cinnamaldehyde Loaded on Halloysite Nanotubes

**DOI:** 10.3390/foods10061150

**Published:** 2021-05-21

**Authors:** Rui Cui, Bifen Zhu, Jiatong Yan, Yuyue Qin, Mingwei Yuan, Guiguang Cheng, Minglong Yuan

**Affiliations:** 1 Institute of Agriculture and Food Engineering, Kunming University of Science and Technology, Kunming 650550, China; cuirui910@163.com (R.C.); yi199602@163.com (B.Z.); 17854117210@163.com (J.Y.); ggcheng@kmust.edu.cn (G.C.); 2 Engineering Research Center of Biopolymer Functional Materials of Yunnan, Yunnan Nationalities University, Kunming 650550, China; yml@vip.163.com

**Keywords:** cinnamaldehyde, halloysite nanotubes, sodium alginate, release

## Abstract

The worsening environment and the demand for safer food have accelerated the development of new food packaging materials. The objective of this research is to prepare antimicrobial food packaging film with controlled release by loading cinnamaldehyde (CIN) on etched halloysite nanotubes (T-HNTs) and adding it to sodium alginate (SA) matrix. The effects of T-HNTs-CIN on the physical functional properties and antibacterial activity of the film were systematically evaluated, and the release of CIN in the film was also quantified. Transmission electron microscopy and nitrogen adsorption experiments showed that the halloysite nanotubes had been etched and CIN was successfully loaded into the T-HNTs. The addition of T-HNTs-CIN significantly improved the water vapor barrier properties and tensile strength of the film. Similarly, the presence of T-HNTs-CIN in the film greatly reduced the negative effects of ultraviolet rays. The release experiment showed that the diffusion time of CIN in SA/T-HNTs-CIN film to fatty food simulation solution was delayed 144 h compared with that of SA/CIN film. Herein, the antibacterial experiment also confirmed the controlled release effect of T-HNTs on CIN. In conclusion, SA/T-HNTs-CIN film might have broad application prospects in fatty food packaging.

## 1. Introduction

In recent years, the need to coordinate the growing environmental pollution problems with technological progress has become very urgent [1]. Hence, the use of biodegradable polymer materials to develop functional food packaging materials has been widely concerned and studied, in order to reduce environmental pollution and meet people’s needs for active food packaging [2,3]. Among many biopolymers, alginate, as a carbohydrate, is often used as a raw material for biodegradable food packaging, which has the characteristics of low cost, good biocompatibility, and excellent film-forming properties [4,5]. Sodium alginate (SA) is a naturally linear water-soluble polysaccharide extracted from brown algae, which is composed of *β*-d-mannuronic acid and *α*-l-guluronic acid (1–4) linking units [6,7]. SA has been widely used in the field of food packaging and biomedical fields because of its low price, easy access, and easy processing. As a food packaging material, SA has advantages, such as good mechanical properties and high transparency [8,9].

Pathogenic microorganisms can cause food spoilage and food-borne diseases, as well as consumers’ concerns about chemical residues in food, making the food industry more and more concerned about the research of natural antibacterial agents in food packaging [10,11]. In order to maximize the function of packaging, extend the use period of food and protect consumers from the threat of food-borne disease outbreaks, antibacterial active substances (such as essential oils) are usually added to increase its function [12]. Cinnamon essential oil is widely used in the field of food, which can be used to protect food without causing harm to human health. There have been many studies that add cinnamon essential oil to food packaging as an antimicrobial agent for food preservation [13,14]. Cinnamaldehyde (CIN) has been approved by the Food and Drug Administration (FDA) and can be used in food products. It is the main ingredient extracted from cinnamon essential oil, which is not only harmless, but also has a broad spectrum of antibacterial and antifungal activities [15]. However, it must be taken into account that CIN is liquid and volatile at room temperature, which is a severe challenge we are currently facing. In this regard, there have been many solutions, such as using porous and other special structure carriers to load active compounds or encapsulating the active substance in chitosan or cyclodextrin [16,17]. In this context, it is one of the most convenient strategies to load CIN with nanoparticles of special structure, which can not only reduce CIN volatilization losses, but also impart the characteristics of nanoparticles to the food packaging system.

Among many nanoparticle carriers, halloysite nanotubes (HNTs) have aroused great interest of researchers due to their ability to trap, protect, and control the release of active substances [18]. HNTs, whose chemical formula is Al_2_Si_2_O_5_(OH)_4_.nH_2_O, which is a subgroup of kaolin. In addition, HNTs have been listed by the FDA as a safe food packaging material [19]. HNTs can be used as a carrier for drug delivery in the medical field because of their unique hollow tubular structure, low cost, and good biocompatibility [12,20]. In particular, HNTs have been shown to have higher adsorption capacity than montmorillonite [21]. Moreover, it has been shown that the addition of HNTs as fillers can improve the barrier and mechanical properties of polymers [10].

The purpose of this study is to develop SA antibacterial composite film with controlled release. The lumen of the original HNTs is limited in volume, and the enlargement of the pores will allow more active chemicals to be loaded. Therefore, in this study, HNT were etched with sulfuric acid to further increase its drug loading, and then CIN was loaded on acidified nanoparticles (T-HNTs) to prepare composite film by method of solution casting. The effects of T-HNTs loaded with CIN on the microstructure, barrier performance, transparency, and antibacterial properties of the composite film were studied, and the release behavior of CIN in the composite film was also investigated.

## 2. Material and Methods

### 2.1. Materials

Sodium alginate (SA) was obtained from Zhejiang Yinuo Biotechnology Company (Lanxi, China). Halloysite nanotubes (HNTs) were purchased from Xi’an Mingda Biotechnology Co., Ltd. (Xi’an, China), with a purity of 99.96%. Sigma-Aldrich (St. Louis, MO, USA) provided the use of glycerin (MW = 92.09). Cinnamaldehyde (CIN) (Purity 98%) was purchased from Shanghai Macklin Biotech Co., Ltd. (Shanghai, China) The manufacturer of the analytical pure sulfuric acid was the Xilong Chemical Factory in Shantou, Guangdong. The other reagents used in this experiment were all of analytical grade.

### 2.2. Etching of HNTs

The etching of HNTs was mainly based on the method of Abdullayev et al., and some changes were made in the process of implementation [22]. Five grams of HNTs were added to 500 mL of sulfuric acid (1.0 M) and the dispersion was stirred and kept at 60 °C for 30 h. The treated HNTs (T-HNTs) were then washed with deionized water five times until their pH range was between 6 and 7. Finally, the samples were dried at 50 °C for 24 h and crushed into powder with a mortar.

### 2.3. Fabrication of T-HNTs-CIN Nanoparticles

The method of T-HNTs loading CIN was mainly modified and implemented by referring to the method of Zou et al. [23]. T-HNTs and CIN were mixed in 30 mL anhydrous ethanol solution, stirred overnight, and then treated with ultrasound for 15 min. The suspension was vacuumed twice for 15 min to remove air to encapsulate CIN into the cavity of HNT. Finally, the suspension was centrifuged to obtain CIN-loaded nanoparticles (T-HNTs-CIN) and dried at room temperature for 24 h.

### 2.4. Preparation of the SA Composite Film

The nanocomposite film was prepared by the solution casting method. First, 2 g sodium alginate powder was dissolved in 100 mL of distilled water containing glycerol (30 wt% relative to SA) and bathed in water at 50 °C for 30 min to prepare the sodium alginate solution. SA/T-HNTs-CIN dispersion was obtained by dispersing the T-HNTs-CIN nanoparticles in sodium alginate solution by loading a certain amount of CIN on T-HNT (5% by weight relative to SA). Simultaneously, the same amount of T-HNTs (5% by weight relative to SA) or CIN (The amount of CIN supported by 5 wt% T-HNTs) was added to the sodium alginate as the control. The prepared dispersions were poured into the polytetrafluoroethylene plate and dried at room temperature for 36 h to form the film. Then, 1% *w*/*v* CaCl_2_ solution was poured onto the film so that the film was completely covered by the solution. After the reaction for one minute, the CaCl_2_ solution was poured out and washed twice with distilled water. Finally, after drying for 48 h at room temperature, it was stored in a desiccator at 25 °C and 50% relative humidity. The manufactured films were identified as SA, SA/CIN, SA/T-HNTs, and SA/T-HNTs-CIN, respectively.

### 2.5. Characterization of Nanoparticles

Field emission transmission electron microscopy (TEM, Tecnai G2 F30 S-Twin) was used to photograph the morphological differences between HNTs and etched HNTs. The nanoparticles were first dispersed in an ethanol solution ultrasonically for 5 min, and then dropped on the surface of the copper grid to be observed after drying.

The change in the porous structure of the nanoparticles was measured by the Micromeritics ASAP2020 system. Before the experiment, the sample was vacuum degassed at 250 °C for 8 h. The specific surface area and pore size distribution of the sample were determined according to the Brunauer Emmett Teller (BET) method and the Barret-Joyner-Halenda (BJH) method, respectively.

### 2.6. Film Thickness

The thickness of the prepared film was measured by randomly selecting five positions with a micrometer (Ningbo Deli Co., Ltd., Ningbo, China), and the accuracy was 0.001 mm.

### 2.7. Microscopic Images

The surface of the SA composite film sample was plated with a layer of gold, and then placed in a vacuum environment to observe the surface morphology of the prepared sample using a field emission scanning electron microscope (SEM, NOVA NANOSEM-450, FEI Co., Ltd., Hillsboro, OR, USA)

### 2.8. Water Vapor Permeability (WVP)

The ability of SA film to block water vapor was measured referring to ASTM E96-95 standard method. Twelve grams of anhydrous silica were put into the weighing cup. The prepared film was cut to a uniform size to seal the rim of the measuring cup. Finally, the weighing cup was placed in a desiccator at 25 °C and 50% relative humidity. The samples were placed in a desiccator to measure the weight of the weighing cup at an interval of 1 h for a total of 12 h, and three samples were set in parallel for each type.

### 2.9. Mechanical Properties

The mechanical properties of the SA composite film are measured by using a microcomputer electronic tensile testing machine (QLW-5E, Xiamen Qunlong Instrument Co., Ltd., Xiamen, China) to obtain its tensile strength and elongation at break. The stretching speed parameter of the equipment was set at 50 mm/min. The determination of each type of film was repeated five times, and each repetition was from a different sampling unit.

### 2.10. Light Transmittance and Opacity of Film

The transmittance of SA composite film in ultraviolet (UV) and visible light region was determined by double beam ultraviolet spectrophotometer. The detailed determination method was based on Achachlouei et al. [24]. The film sample was cut into rectangular pieces and installed in a quartz cuvette for spectrum measurement. The wavelength of measurement was selected as 200–800 nm. The opacity of the film can be calculated by Equation (1).

(1) Opacity=(lg(1/T))/d

where T is the light transmittance of the film at 600 nm, and d is the thickness of the sample. The measurements were repeated three times for each type of film.

### 2.11. The Sustained-Release of CIN in Film in Food Simulants

The CIN release from the SA nanocomposite film was assessed following a method adapted from Muller et al. [25]. Sodium alginate is a hydrophilic colloid. When the sodium alginate composite film is in contact with water or aqueous solution, its matrix structure will change. Therefore, isooctane was selected as the food simulant in this experiment. The film, weighing about 2 g, was immersed in 1 L of isooctane solution at 20 °C. A total of 1 mL solution was taken out from the food simulation solution at certain intervals (1 mL of isooctane solution was supplemented after taking it out), and the content of CIN in the solution was measured by a dual-beam UV-Vis spectrophotometer with a wavelength of 279 nm. At the same time, SA/CIN and SA/T-HNTs-CIN corresponding film without CIN were used as controls, and three parallel films were set for each sample. The cumulative release rate of CIN in the film can be calculated by Equation (2).

(2) $$C\;\left(\%\right)=(C\times V\times k/C_{0}\times m)\times 100$$

where C is the cumulative release rate of CIN (%), C is the concentration of CIN in isooctane at t (mg/mL), V is the volume of isooctane solution (mL), k is the dilution factor of the solution, $C_{0}$ is the initial concentration of CIN (mg/mL), and m is the amount of film (mg).

### 2.12. In Vitro Antibacterial Activity of the Film in the Release Experiment

In order to assess the antimicrobial properties of the manufactured film, a slight modification of the previous method was used [26]. *Staphylococcus aureus* (*S. aureus*) and *Escherichia coli* (*E. coli*) (both of which were provided by the Microbiology Laboratory of the Faculty of Agriculture and Food Engineering, Kunming University of Science and Technology, Yunnan, China) were stored at −80 °C. The specific operation process of the antibacterial experiment was as follows. First, the long-term stored *S. aureus* and *E. coli* were inoculated with tryptic soy broth (TSB) medium. This step was then repeated to activate the activity of both strains. Then, 100 μL of the activated bacterial solution was added to a 10 mL TSB medium containing 0.2 g of sample, so that the bacterial solution concentration in the medium was about 10^5^ CFU/mL. Subsequently, the bacterial solution containing the sample was cultured on a shaker for 12 h, the temperature was set to 37 °C, and the speed was 180 rpm. After the time was up, the bacterial liquid was evenly diluted and spread in tryptic soy agar medium and placed in an incubator at 37 °C. After 18–24 h of colony culture, the number of colonies was calculated. In particular, the sample was regularly taken out of the food simulant and wiped dry for the antibacterial test during the release experiment. Each sample was repeated for three times.

### 2.13. Statistical Analysis

The data were analyzed by one-way analysis of variance (ANOVA) using SPSS 21.0 (Chicago, IL, USA), followed by Duncan’s multiple comparison test at 95% confidence level.

## 3. Results and Discussion

### 3.1. Characterization of Nanoparticles

**Figure 1:**
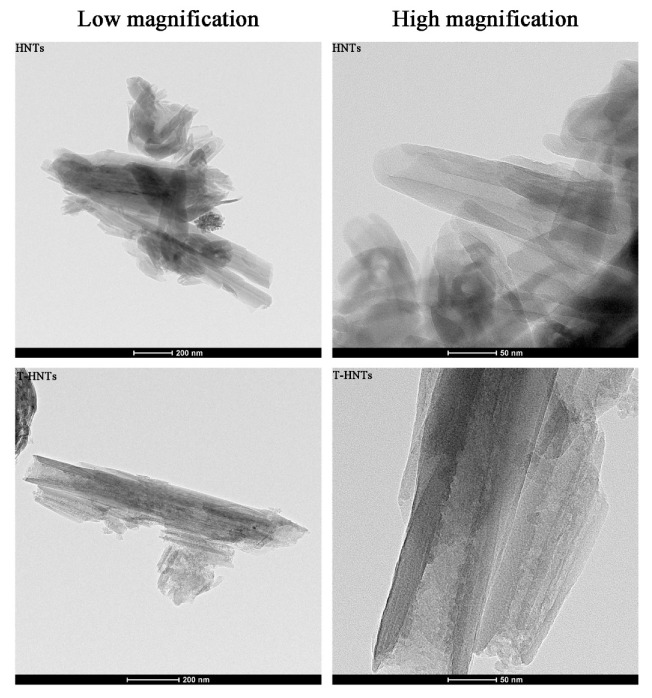
TEM images of HNTs and T-HNTs.

TEM images of the pristine HNTs and the treated HNTs nanoparticles are shown in Figure 1. Both HNTs and T-HNTs are cylindrical, with a central transparent region extending lengthwise along the nanotubes, indicating that the HNTs are hollow and open. No significant changes are observed in the tube length of HNTs and T-HNTs. Interestingly, after HNTs were treated with sulfuric acid, the inner lumen was clearly etched, and their inner diameters were significantly increased by about 10–20 nm. This indicated that sulfuric acid treatment resulted in an increase in the inner diameter of HNTs.

The porous structure parameters of HNTs, T-HNTs, and T-HNTs-CIN are shown in Table 1. After HNT was etched by sulfuric acid, its specific surface area increased from 25.50 to 76.61 m^2^/g, and its pore volume increased from 0.30 to 0.39 m^3^/g, which were consistent with the TEM observation results. The same trend was found for average pore size of HNTs. The selective dissolution of the AlO_6_ octahedral layer in the inner cavity of HNTs and the decomposition of silica would cause the increase of the specific surface area and pore volume of HNTs [27]. Garcia-Garcia et al. found the same trend when treating halloysite nanotubes with acid [28]. CIN itself is volatile, and samples need to be degassed before measurement, which will cause a certain error between the actual measurement result and the theoretical value (the actual measurement result is less than the theoretical value). If the CIN was not successfully loaded onto the T-HNTs, the specific surface area of the T-HNTs-CIN did not change significantly. The actual results showed that the specific surface area and pore volume of the T-HNTs-CIN increased compared with the HNT, but decreased compared with the T-HNTs, indicating that the CIN was successfully loaded onto the T-HNTs.

### 3.2. Surface Morphology of Composite Film

**Figure 2:**
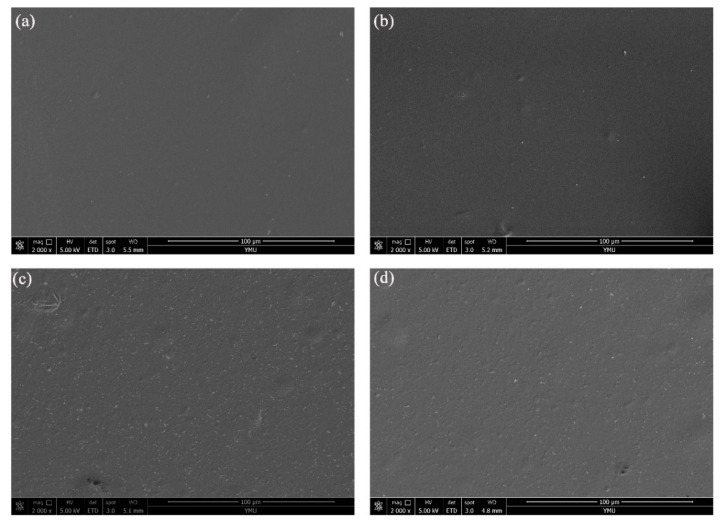
SEM micrographs of the surface of the bio-nanocomposite film: (**a**) SA film, (**b**) SA/CIN film, (**c**) SA/T-HNTs film, and (**d**) SA/T-HNTs-CIN film.

Figure 2 illustrates the surface morphology of four different formulations of SA film. Figure 2a is the SA film, the surface of which is uniform and smooth. The presence of CIN in the SA matrix did not significantly affect the surface of the matrix. However, when T-HNTs were added to the SA matrix, many uniformly distributed white spots appeared on the surface of SA/T-HNTs film. Similarly, SA/T-HNTs-CIN film showed the same phenomenon. This implied that there was no significant difference between T-HNTs and CIN-loaded T-HNTs on the surface morphology of the SA film.

### 3.3. WVP

The key factor in evaluating the feasibility of composite materials in food preservation is to determine the WVP value of composites. During food preservation, edible or biodegradable film can be used to reduce the transfer of moisture from the environment to the inside of the package [29]. The WVP values of neat SA film, as well as the SA nanocomposite film, are shown in Table 2. The WVP value of SA film is 38.2 × 10^−2^ g mm/h KPa m^2^. The barrier of SA composite film to water vapor was significantly enhanced after incorporation of CIN or T-HNTs. When CIN was immobilized on T-HNTs and then incorporated into SA matrix, the WVP value of SA/T-HNTs-CIN film was reduced by 14.7% compared with that of SA film. There are many factors that affect the barrier properties of the film, such as the hydrophobicity, the structure, and the compatibility of the material. CIN is inherently hydrophobic, and its addition might increase the hydrophobic/hydrophilic ratio of the film [30]. As a high aspect ratio aluminosilicate mineral, HNTs have barrier properties to water vapor. HNTs dispersed in the matrix might make the path of water molecules through the matrix become tortuous [10]. All of these might be the reasons for the decrease of WVP value of the film. Yousefi et al. added halloysite nanotubes and *Origanum vulgare* essential oil to the film matrix, which also improved the water resistance of the film [29].

### 3.4. Mechanical Properties

The effect of T-HNTs nanoparticles loaded with CIN on the mechanical properties of SA composite film was studied. As presented in Table 3, it was evident that with the addition of T-HNTs-CIN nanoparticle, the tensile strength (TS) of the SA/T-HNTs-CIN nanocomposite film improved, and its TS value was increased by 20.8% compared to the SA film. This was similar to the effect of halloysite nanotubes on the mechanical properties of carrageenan/gelatin films previously published by Akrami-Hasan-Kohal et al. [31]. The potential strain-induced arrangement of the clay particle layer in the polymer matrix and the interaction between the polymer and the hydrogen bonds in the clay minerals might contribute to the improvement of the tensile properties of the film [32,33]. The elongation at break (*ε*) of the film was not changed significantly by the T-HNTs-CIN nanoparticle addition. However, the addition of CIN significantly increased the flexibility of the film. Ahmed et al. also reported that adding clove essential oil to the film matrix increased the flexibility of the film [34]. The presence of the CIN in the SA matrix might hinder the polymer–polymer intermolecular attraction [35]. However, CIN was added to the SA matrix after being loaded by T-HNTs, and most of the CIN was present in the T-HNTs, thereby reducing the plasticizing effect of CIN.

### 3.5. Light Transmittance and Opacity of Film

**Figure 3:**
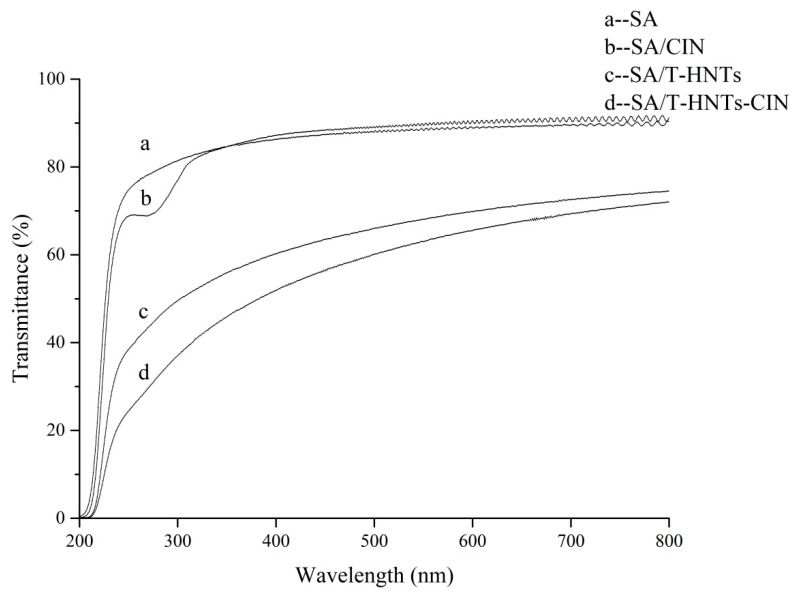
Light transmittance of the nanocomposite film: (**a**) SA film, (**b**) SA/CIN film, (**c**) SA/T-HNTs film, and (**d**) SA/T-HNTs-CIN film.

The UV-visible spectrum for SA nanocomposite film is displayed in Figure 3. It can be seen that SA film exhibited high light transmittance in both UV and visible regions, especially in the 300–800 nm region with a high light transmittance of more than 80%. The light transmittance of SA/CIN film at 240–300 nm was significantly lower than that of SA film. The anti-UV effect of CIN might be its own aromatic compound, and its chemical bond could absorb UV light [26]. Ahmed et al. had previously found a similar phenomenon in the study of polylactide/cinnamon oil composite films [36]. Moreover, as can be seen from curves c and d in Figure 2, the transmittance of the composite film at all wavelengths was greatly reduced after the addition of T-HNTs in SA film.

Table 4 shows the transmittance values of the composite films at 240 (UV-C), 300 (UV-B), 360 (UV-A), and 600 nm (visible light) as well as the opacity of different formulation film. The light transmittances of SA film at 240, 300, 360, and 600 nm are 69.5, 81.8, 85.0, and 87.4%, respectively, and the transmittance value after the addition of T-HNTs-CIN decreased to 22.8, 40.9, 50.1, and 66.1%, respectively. This might be due to the combined action of T-HNTs and CIN. The presence of T-HNTs in the film matrix might block or diffract the light, thus affecting the transmittance of light at all wavelengths [8]. Huang et al. also found a similar phenomenon when halloysite was added during the preparation of agar-based nanocomposite films [37]. The transparency of SA composite film decreased with the addition of HNTs. At the same time, the color of the film was whitened by the addition of the white powder HNTs. It is noteworthy that the addition of T-HNTs-CIN resulted in a greater reduction in the transmittance of UV light than that of visible light. This means that by preparing the composite film with T-HNTs-CIN, the UV barrier properties can be improved without sacrificing the transparency of SA film. The nanocomposite film with high ultraviolet shielding performance has high application potential as a transparent ultraviolet blocking packaging material.

### 3.6. Slow-Release Behavior of the CIN in Food Simulants

**Figure 4:**
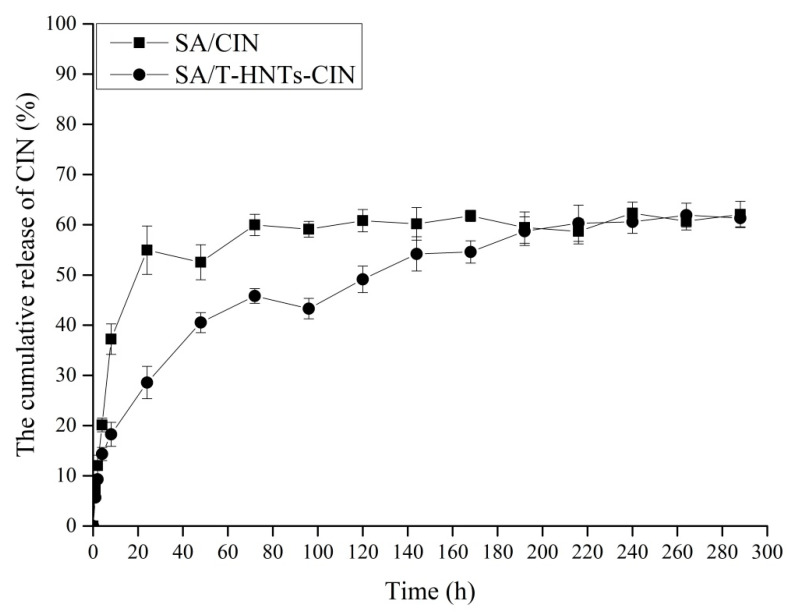
The cumulative release of CIN in the nanocomposite film: SA/CIN film and SA/T-HNTs-CIN film.

Isooctane was used as food simulant to simulate food with hydrophobic fats. The cumulative release of CIN by the SA film without T-HNTs and the SA film with T-HNTs in food simulant are shown in Figure 4.

The whole process of the release experiment was carried out under stable environmental conditions (temperature of 20 °C, relative humidity 75%). The SA/CIN and SA/T-HNTs-CIN film in food simulant released most of CIN in the first 24 h, and the cumulative release of SA/CIN film was higher (58.95%), which was significantly higher than SA/T-HNTs-CIN film (28.57%). Subsequently, the release rate of CIN in the two films slowed down significantly. Finally, the release of CIN from SA/CIN film reached a peak of 59.97% at 72 h, and the release amount of CIN was about 16.64 mg, while the release of SA/T-HNTs-CIN film reached a stable level at 216 h, and the cumulative release of film stabilized at 60.31% (The release of CIN was about 17.44 mg). The presence of T-HNTs in SA/T-HNTs-CIN film slowed down the release rate of CIN, which in turn delayed the time when CIN reached stability. Shen et al. prepared novel sodium alginate-based double network hydrogel spheres after loading urea on HNTs, which reduced the release rate of urea [38]. The release of CIN in the SA composite film matrix into the food simulant is affected by many factors. First, the liquid molecules in the solvent diffuse from the outer surface of the film into the matrix of the SA composite film. Then, the polymer matrix network relaxes due to the presence of the solvent in the film matrix. Lastly, CIN is released from the relaxed polymer matrix into the food simulating liquid until the thermodynamic equilibrium between the SA composite film and the food simulating liquid is reached. Of course, the last step is influenced not only by mass transfer, but also by the interaction between the volatile compounds and the matrix [25,39].

According to the steps described above for the release of the active compound from the SA composite film matrix, and in combination with the release curves of the two film systems in food simulation solution, it can be seen that the release rate of the SA composite film supported by T-HNTs was significantly slower than that of the SA/CIN film. This observed behavior could be explained by the retarded release of CIN by T-HNTs. The loading of CIN by T-HNTs was mainly the adsorption of intracavity and external surface of T-HNTs to CIN [40]. This increased the mass transfer steps of CIN in the release process, thereby prolonging the release time of CIN. Compared with CIN directly added to SA matrix, CIN was added to SA matrix in a HNTs loaded manner, and CIN was better protected to maintain its activity through the action of HNTs carrier, and the release time of CIN from the controlled release system was prolonged, thus maximizing the function of CIN. Therefore, it can be concluded that the presence of HNTs in SA/T-HNTs-CIN film controlled release system could effectively alleviate the initial burst release of CIN and prolong the action time of CIN. The SA/T-HNTs-CIN film slow-release system might have a good application prospect in the packaging of fatty foods.

### 3.7. In Vitro Antibacterial Activity of the Film in the Release Experiment

**Figure 5:**
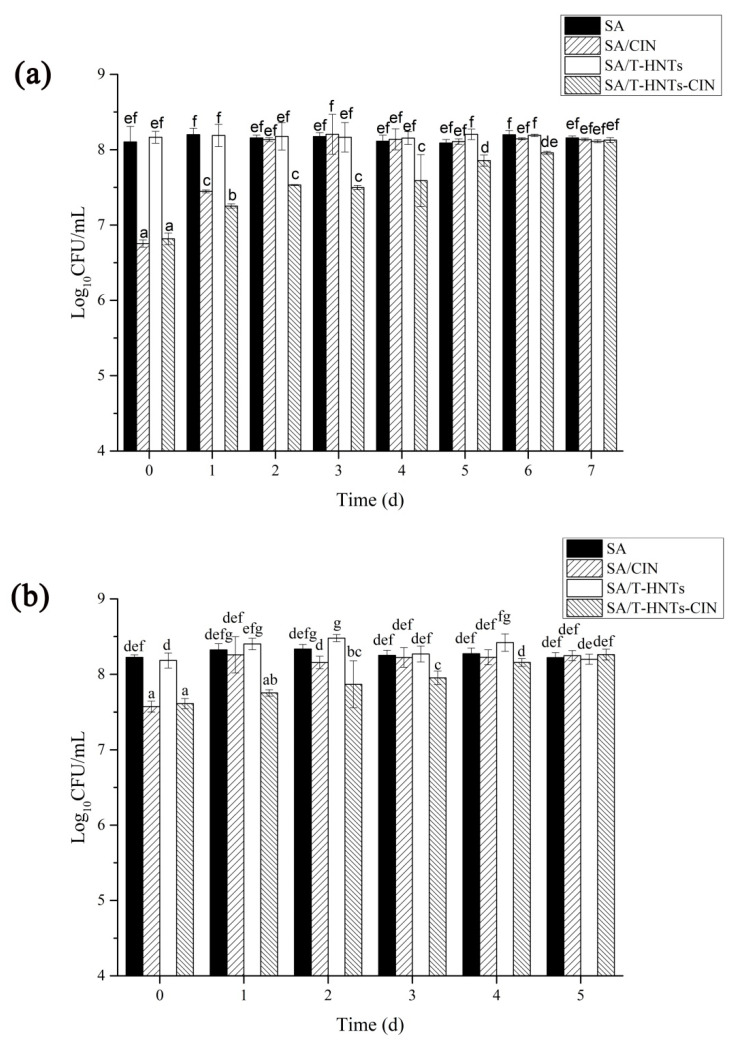
Antimicrobial activity of the composite film: (**a**) *Staphylococcus aureus* and (**b**) *Escherichia coli.* Values followed by different letters (a–f) were significantly different (*p* < 0.05), where a is the lowest value.

The effects of the manufactured film on the antibacterial activity of typical food-borne pathogens (*S. aureus* and *E. coli*) were investigated, and the results are shown in Figure 5. As expected, the presence of T-HNTs in the film matrix has no antibacterial activity against *S. aureus* and *E. coli.* However, on day 0 of the experiment, SA/CIN and SA/T-HNTs-CIN film decreased by 1.34 and 1.35 Log_10_CFU/mL, respectively, compared to the control group. This might be attributed to the presence of CIN in the polymer matrix. A large number of previous studies have shown that CIN had inhibitory effects on the growth of a variety of bacteria, because CIN might damage the cell membrane of bacteria, leading to changes in cytoplasmic leakage and membrane permeability [41,42]. Similarly, for *E. coli*, the number of colonies in the film containing CIN was significantly reduced by 0.65 and 0.57 Log_10_CFU/mL, respectively, compared with the control group. Interestingly, we found that *S. aureus* was more susceptible to CIN inhibition than *E. coli.* This phenomenon could be attributed to the fact that the cell wall of *E. coli* (Gram-negative bacterium) has an extra layer of lipopolysaccharide outer membrane than that of *S. aureus* (gram-positive bacterium). The lipopolysaccharide outer membrane has a good barrier effect on hydrophobic substances (CIN). Although it cannot completely block hydrophobic compounds, it limits the penetration of CIN into microbial cells and reduces the inhibitory effect [43].

In addition, with the increase of release time, the bacteriostatic effect of the film containing CIN on the two kinds of bacteria became less and less. There was no significant difference between SA/CIN film and SA in the colony count of *S. aureus* on the second day. However, the SA/T-HNTs-CIN film showed no significant difference in the number of colonies from the SA/T-HNTs film on 7 days. The same trend was observed for *E. coli*, where CIN was fixed by T-HNTs and then added to SA matrix, and the bacteriostatic duration was extended by 4 days compared with SA/CIN films. These results confirmed that the SA/T-HNTs-CIN film had a controlled release effect on CIN, successfully alleviating the interaction between the active compound and the surrounding environment and making it release slowly. This packaging system can be used as a new and promising alternative to renewable food packaging. Of course, future research in this area should focus more on increasing the loading of active substances or using multiple loading systems to improve their applicability.

## 4. Conclusions

In this work, the HNTs were etched and then loaded with CIN, and the food functional packaging film with continuous release and antibacterial activity were prepared by solvent volatilization. It was confirmed that the cavities of HNTs were etched and their specific surface area increased significantly after sulfuric acid treatment. CIN was first loaded by T-HNTs and then added to SA matrix, which greatly improved the mechanical properties of the film and the barrier property of water vapor. In addition, the combined effect of T-HNTs and CIN could increase the UV shielding effect while losing less transparency. In the release experiment, the SA/T-HNTs-CIN film could be released continuously in the fatty food simulation solution for 216 h, which was 144 h longer than that of SA/CIN film. The antibacterial activity of SA/T-HNTs-CIN film against *S. aureus* and *E. coli* was longer by five days and four days than that of SA/CIN film without T-HNTs.

## Figures and Tables

**Table 1:** The surface area, pore volume, and average pore size of samples.

Samples	S_BET_ (m^2^/g)	Pore Volume (cm^3^/g)	Average Pore Size (nm)
HNTs	25.50	0.30	1.88
T-HNTs	76.61	0.39	2.41
T-HNTs-CIN	51.39	0.37	2.13

**Table 2:** Water vapor permeability of SA-based composite film.

Films	Thickness (mm)	WVP (×10^−2^ g mm/24 h·KPa·m^2^)
SA	0.030 ± 0.001 ^a^	1.59 ± 0.05 ^b^
SA/CIN	0.033 ± 0.004 ^a^	1.31 ± 0.15 ^a^
SA/T-HNTs	0.035 ± 0.001 ^a^	1.41 ± 0.02 ^a^
SA/T-HNTs-CIN	0.033 ± 0.003 ^a^	1.36 ± 0.11 ^a^

Data are presented as mean ± standard deviation and different letters (a, b) within the columns shows the significant differences (*p* < 0.05), where ^a^ is the lowest value.

**Table 3:** Mechanical properties of SA nanocomposite film.

Film	TS (MPa)	*ε* (%)
SA	66.4 ± 4.28 ^a^	2.76 ± 0.30 ^a^
SA/CIN	70.4 ± 2.45 ^ab^	3.35 ± 0.07 ^b^
SA/T-HNTs	77.3 ± 7.50 ^bc^	2.66 ± 0.09 ^a^
SA/T-HNTs-CIN	80.2 ± 3.81 ^c^	2.97 ± 0.30 ^ab^

Data are presented as mean ± standard deviation and different letters (a–c) within the columns shows the significant differences (*p* < 0.05), where ^a^ is the lowest value.

**Table 4:** Transmittance (%) and opacity and values of nanocomposite film in the visible, UV-A, UV-B, and UV-C regions.

Film Sample	UV-C (240 nm) T (%)	UV-B (300 nm) T (%)	UV-A (360 nm) T (%)	Visible (600 nm) T (%)	Opacity (AU. nm/mm)
SA	69.5 ± 2.29 ^d^	81.8 ± 0.67 ^d^	85.0 ± 0.32 ^c^	87.4 ± 2.45 ^c^	2.50 ± 0.21 ^a^
SA/CIN	64.4 ± 1.13 ^c^	76.1 ± 1.13 ^c^	84.8 ± 0.57 ^c^	89.9 ± 0.85 ^c^	2.00 ± 0.10 ^a^
SA/T-HNTs	35.6 ± 1.62 ^b^	50.2 ± 1.07 ^b^	57.5 ± 0.79 ^b^	70.3 ± 0.50 ^b^	4.54 ± 0.55 ^b^
SA/T-HNTs-CIN	22.8 ± 2.40 ^a^	40.9 ± 4.62 ^a^	50.1 ± 3.27 ^a^	66.1 ± 0.57 ^a^	4.78 ± 1.15 ^b^

Data are presented as mean ± standard deviation and different letters (^a^–^d^) within the columns shows the significant differences (*p*< 0.05), where ^a^ is the lowest value.
